# A comprehensive review and comparison of L-tryptophan biosynthesis in *Saccharomyces cerevisia*e and *Escherichia coli*

**DOI:** 10.3389/fbioe.2023.1261832

**Published:** 2023-12-04

**Authors:** Xinru Ren, Yue Wei, Honglu Zhao, Juanjuan Shao, Fanli Zeng, Zhen Wang, Li Li

**Affiliations:** ^1^ College of Science and Technology, Hebei Agricultural University, Cangzhou, China; ^2^ College of Life Sciences, Hebei Agricultural University, Baoding, China; ^3^ Hebei Key Laboratory of Analysis and Control of Zoonotic Pathogenic Microorganism, Baoding, China

**Keywords:** L-tryptophan, *Saccharomyces cerevisiae*, *Escherichia coli*, biosynthesis, stress fitness

## Abstract

L-tryptophan and its derivatives are widely used in the chemical, pharmaceutical, food, and feed industries. Microbial fermentation is the most commonly used method to produce L-tryptophan, which calls for an effective cell factory. The mechanism of L-tryptophan biosynthesis in *Escherichia coli*, the widely used producer of L-tryptophan, is well understood. *Saccharomyces cerevisiae* also plays a significant role in the industrial production of biochemicals. Because of its robustness and safety, *S. cerevisiae* is favored for producing pharmaceuticals and food-grade biochemicals. However, the biosynthesis of L-tryptophan in *S. cerevisiae* has been rarely summarized. The synthetic pathways and engineering strategies of L-tryptophan in *E. coli* and *S. cerevisiae* have been reviewed and compared in this review. Furthermore, the information presented in this review pertains to the existing understanding of how L-tryptophan affects *S. cerevisiae’s* stress fitness, which could aid in developing a novel plan to produce more resilient industrial yeast and *E. coli* cell factories.

## Introduction

L-tryptophan (L-Trp) is an essential aromatic amino acid required for protein synthesis in humans and animals (Modoux et al., 2021). In recent years, the application of L-Trp in food additives and feed and in medical fields has attracted much attention (Xiao et al., 2023). L-Trp is typically produced through chemical synthesis, direct fermentation, and enzymatic conversion (Liu et al., 2012). However, with the advent and expansion of the green business, chemical synthesis has become less desired. Enzymatic conversion is an efficient method for manufacturing L-Trp, but its high cost is its main downside. Microbial fermentation is often favored over chemical synthesis and enzymatic conversion processes because it allows for the environmentally benign synthesis of L-Trp from inexpensive and renewable carbon sources (Niu et al., 2019). Then, a pressing issue for the growth of this industry is how to enhance the yield of L-Trp through the microbial fermentation process. Although the production of L-Trp has significantly grown in recent years due to the ongoing development of superior genome sequencing and synthetic biology technologies, methods for maximizing L-Trp yield still need to be refined (Liu et al., 2022).

The metabolic engineering alteration of core strains is one of the essential components of microbial fermentation. *Escherichia coli* (*E. coli*) and *Corynebacterium glutamicum* (*C. glutamicum*) are well-studied chassis cell factories for producing L-Trp (Wendisch et al., 2006; Guo et al., 2022). *Saccharomyces cerevisiae* (*S. cerevisiae*) is a prominent representative among eukaryotic microbial hosts, and aromatic amino acid metabolism by yeast is of interest for some industrial applications (Luttik et al., 2008b). *S. cerevisiae* has also been utilized to produce L-Trp and its derivativesin bioprocessing. This choice is attributed to the organism’s resilience and enhanced ability to withstand challenging fermentation conditions. Additionally, *S. cerevisiae* can express P450 oxidase in eukaryotic secondary metabolic pathways, further supporting its suitability (Zhang et al., 2016). The metabolic engineering of the L-Trp biosynthetic pathway has emerged as a research hotspot to generate the L-Trp overproducing microbial strains for industrial production of L-Trp (Niu et al., 2019). However, additional investigation is required in order to elucidate the metabolic pathways and ascertain the specific targets for alteration.

Another way to enhance L-Trp production is to make fermentation strains more resilient and adapted to the environment. During fermentation, *E. coli* and *S. cerevisiae* are exposed to various environmental stresses, such as osmotic, ethanol, oxidation, and heat stress, which can seriously affect the growth and metabolism of microbes (Shen et al., 2022). Studies have shown increasing interest in studying amino acid metabolism related to signaling and metabolic adaptation to stress in plants and microbes in recent years (Zeng et al., 2022). To improve the robustness of *E. coli*, strategies such as adaptive laboratory evolution and genome-wide evolutionary engineering are commonly used (Yao et al., 2022). Whether *E. coli* tolerance to stressful settings can be improved by regulating L-Trp to expand L-Trp production capacity further has received less attention, with most studies focused on regulating L-Trp for stress adaption in *S. cerevisiae*. Exogenous L-Trp addition and modulation of overexpression or downregulation of L-Trp-related genes were discovered to successfully improve *S. cerevisiae* adaptability to environmental stress and boost L-Trp synthesis (Kavšček et al., 2015).

The biosynthetic pathway and the main regulatory mechanisms involved in L-Trp production in *E. coli* and *S. cerevisiae* have been reviewed and compared in this review. In addition, the latest strategies used to enhance the production of L-Trp in *E. coli* and *S. cerevisiae* have been summarized. Lastly, the relationship between L-Trp and the stress tolerance of *S. cerevisiae* has been highlighted, along with a discussion of the current strategies for regulating the metabolism of amino acids, especially L-Trp, to improve the robustness of *S. cerevisiae* and *E. coli*.

## The synthetic pathways of L-Trp

**FIGURE 1:**
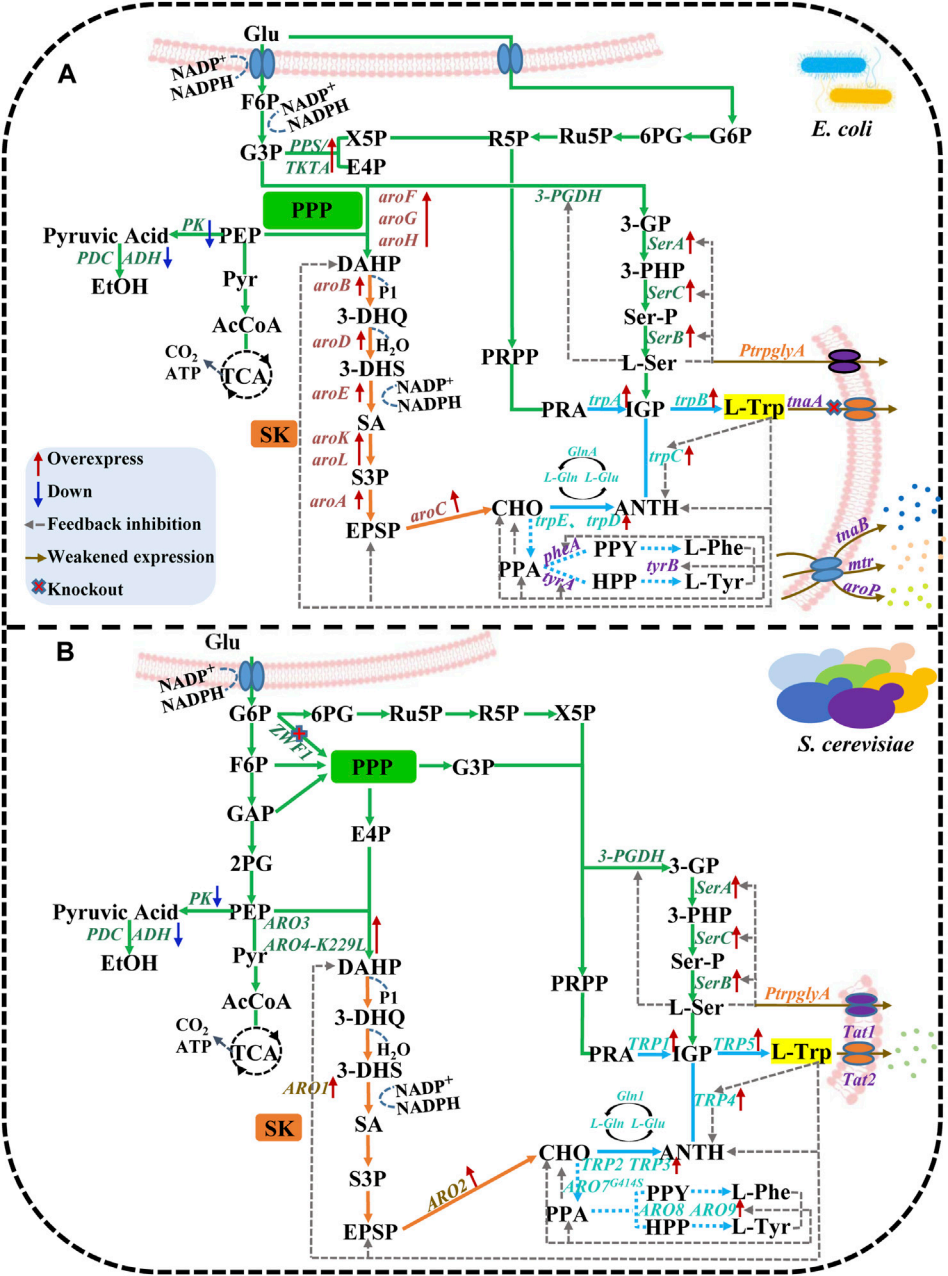
The biosynthetic pathway of L-Trp in *E. coli* and *S. cerevisiae*. **(A)** *E. coli* and **(B)** *S. cerevisiae* are jointly involved in three synthetic pathways: central metabolic, shikimic acid, and chorismate. The red solid line arrow indicates overexpress, the blue solid line arrow indicates down, the gray dotted line arrow indicates feedback inhibition, the brown solid line arrow indicates weakened expression, and × indicates knockout.

Both *S. cerevisiae* and *E. coli* are jointly involved in three synthetic pathways of L-Trp synthesis: the central metabolic pathway, the shikimic acid (SK) pathway, and the chorismate (CHO) pathway. Under oxygen-rich conditions, the Embden-Meyerhof-Parnas pathway transforms a portion of glucose into phosphoenolpyruvate (PEP), while the tricarboxylic acid cycle (TCA) consumes the other portion of glucose (Carvalho et al., 2019). Hexose monophosphate and erythrose-4-phosphate (E4P) are eradicated to generate 3-deoxy-D-arabinogenose-7-phosphate (DAHP). SK pathway starts with DAHP, and the resulting 3-dehydrogenate, 3-dehydrooxalate (3-DHS) and 3-phosphate are converted to 5-enol propyl oxalate 3-phosphate (Gottardi et al., 2017). In the CHO pathway, anthranilate (ANTH) and phenylpyruvate (PPA) are first generated, leading to the production of L-Glutamic acid (L-Glu). ANTH is converted into indoglycerophosphate and anthranilate-5-phosphoribosyl pyrophosphate (PRA), while indole and serine together synthesize L-Trp. Three aromatic amino acids are produced in the CHO pathway: L-Phenylalanine (L-Phe), L-Tyrosine (L-Tyr), and L-Trp. The other two aromatic amino acids, i.e., L-Phe and L-Tyr, negatively affect L-Trp (Tröndle et al., 2020). This study undertakes a comparative analysis of L-Trp biosynthesis and regulation in *E. coli* and *S. cerevisiae*, focusing on three distinct synthetic pathways (Figure 1).

## Regulation of L-Trp production in the key synthetic pathways

### L-Trp regulation in the central metabolic pathway

In *E. coli*, 70% of glucose entering the cell is consumed to produce fructose-6-phosphate (F6P) and glyceraldehyde-3-phosphate (G3P). The remaining glucose enters into the Pentose Phosphate Pathway (PPP) pathway. PEP and E4P are essential precursors of L-Trp synthesis. The L-Trp production can be enhanced by directly upregulating the concentration of these precursors or reducing the competing reactions (Ikeda, 2006). When *E. coli* is grown in a restricted medium with glucose as a carbon source, about 50% of PEP is consumed by the phosphotransferase system (PTS) to carry out glucose transport and phosphorylation (Gosset, 2005). Only 3% of PEP synthesizes DAHP (Gosset, 2005). Low intracellular PEP concentration in *E. coli* leads to decreased carbon fusion in the TCA cycle and PPP pathway, resulting in poor cell growth (Li et al., 2020a). The introduction of the *glf* and *glk* genes encoding glucose transporter and glucokinase, respectively, in exogenous efficient transport systems like *Pseudomonas* is less efficient than the modification of the PTS system, which results in glucose no longer consuming PEP and an increase in intracellular PEP (Escalante et al., 2012). Therefore, PTS mutant strains (*ptsHIcrr* ^*−*^, *glf-glk* ^*+*^, and *ptsG* ^*−*^) were constructed in *E. coli* for batch fermentation (Wu et al., 2017). The growth of *ptsHIcrr* ^*−*^ and *glf-glk* mutant bacteria was up to an OD_600_ value of 125, which was 47% higher than the original strain. The glucose conversion rate was also increased by 26.5%. On the other hand, *ptsG* ^*−*^ mutant bacteria grew up to an OD_600_ value of 100, which was 17.6% higher than the original strain. The glucose conversion rate was also increased by 17.4% (Wu et al., 2017).

In another study, *Glf* in *Z. mobilis* and *Glk* in *E. coli* for glucose utilization were used to replace the PTS system (Tang et al., 2013; Noda et al., 2016; Noda et al., 2017). According to reports, intracellular glucose can undergo phosphorylation by using ATP as a phosphoric acid donor. This process facilitates the integration of *Glf* into the *ptsG* site of SX2 strains, thereby exerting control over the production of *glf* genes. These genes are regulated by promoters exhibiting varying degrees of expression intensity (Lu et al., 2012). Among these promotors, the weak promoters *P*_*M1-12*_ and *P*_*trc*_ *-pyc* ^*P458S*^ overexpressed *glf* and *glk* corresponding to strain SX11, fermented in a 5 L bioreactor for 40 h. SX11 strain produced L-Trp titers as high as 41.7 g/L, yielding 34.1 times than the L-Trp yield in the original strain SX2 (Xiong et al., 2021). Phosphoenolpyruvate synthase (PPS) and transketolase (tktA) are the main enzymes required to form PEP and E4P in *E. coli* directly. By overexpressing *pps* and/or *tktA* in *E. coli*, the expressions of PEP and E4P have been upregulated, resulting in 2 times higher production of DAHP (Shen et al., 2012). Similarly, the upregulation of the citric acid pathway and TCA cycle resulted in the upregulation of *citT*, which transports citric acid into cells. After 24 h of culture, the accumulated L-Trp was 7 g/L (Du et al., 2019). Knocking out the gene encoding PEP to degrade pyruvate kinases *pykA* and *pykF*, as well as blocking the metabolism of PEP, also facilitated the carbon metabolic flow and led to higher aromatics and DAHP yields (Gosset et al., 1996; Meza et al., 2012).

After balancing the effect of PEP on TCA, 5 g/L L-Trp was obtained from *E. coli* strain TRP07 with a glucose yield of 0.186 g/g after 36 h of fermentation in a 5 L bioreactor (Chen et al., 2018b; Du et al., 2019). L-Serine (L-Ser), a precursor in L-Trp synthesis, is often inhibited by D-3-phosphoglycerate dehydrogenase (*3-PGDH*) during synthesis process (Grant, 2018). L-Ser is mostly undersupplied, and thus, L-Trp cannot be overproduced (Chen and Zeng, 2017). However, removal of the *SerA* gene encoding *3-PGDH* regulatory domain can stop the inhibition of L-Ser (Bell et al., 2002).

In *S. cerevisiae*, glucose enters the cell to synthesize glucose-6-phosphate (G6P), F6P, G3P, glyceraldehyde-3-phosphate (GAP), 2-phosphate glycerate (2PG), and finally PEP. Like *E. coli*, *S. cerevisiae* needs to transform the non-oxidized part of PPP to optimize the synthesis of E4P, overexpression of *tkl1* (encoding transketolase isoenzyme 1), and deletion of *Zwf1* (Curran et al., 2013). This resulted in increased carbon flux into PPP at the non-oxidizing node, which further enhanced the flux of glycolysis intermediates F6P and G3P to E4P and X5P by 7 times and increased the 3-dehydroshikimic acid (3-DHS) titer by 7.8 times (Deaner and Alper, 2017). In addition, the Crabtree effect also exists in *S. cerevisiae*. Pyruvate kinase converts the majority of the precursor PEP into pyruvate. Pyruvate is then converted to ethanol by pyruvate decarboxylase and alcohol dehydrogenase (Dai et al., 2018). Previous studies have reported that only 1% of PEP is consumed via the entry into the AAP in *S. cerevisiae* (Blank et al., 2005). However, ^13^C labeled metabolic flux analysis combined with quantitative PCR revealed that expressing the *ARO4*-K229L (*ARO4* allele) could increase the availability of PEP and E4P and reduce the activity of pyruvate kinase or pyruvate decarboxylase, resulting in the accumulation of 17.9 mg/g glucose and 358 mg/L DAHP (Suástegui et al., 2016).

Significant rate-limiting steps in the central metabolic pathway are overcome using various genetic techniques, such as the overexpression and downregulation of crucial enzymes. It is possible to control the carbon flux into L-Trp biosynthesis in the central metabolic pathway (Báez-Viveros et al., 2007) or to increase the availability of the L-Trp biosynthesis precursors PEP and E4P to increase the yield of L-Trp synthesis from glucose (Bongaerts et al., 2001). However, the PPP pathway, where *tktA* is the primary isozyme and overexpression of *tktA* dramatically boosted the yield of aromatic compounds, and consequently, the availability of E4P accounts for a portion of the difference between the pathways for DAHP synthesis in *E. coli* and *S. cerevisiae*. Differences in *tktA* transcript levels between strains may impact L-Trp synthesis (Báez-Viveros et al., 2007).

### Regulation of shikimic acid pathway

**FIGURE 2:**
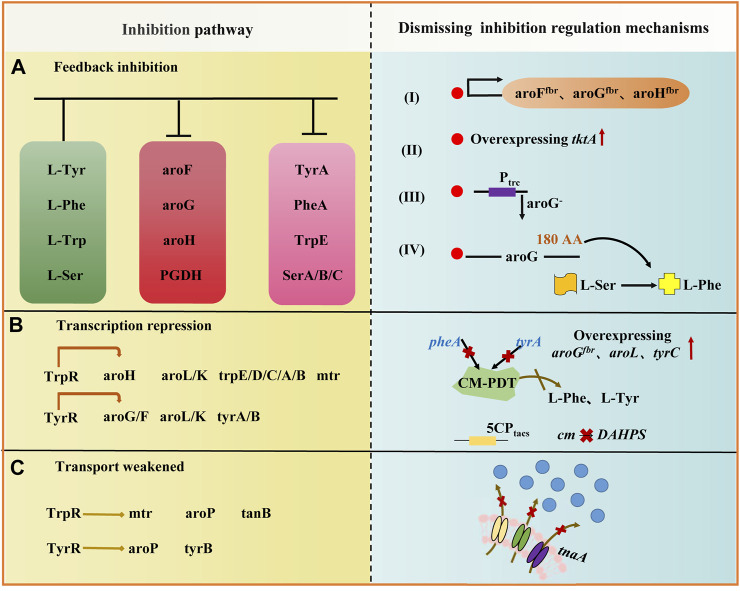
Inhibition pathway and dismissing inhibition regulation mechanism of L-Trp biosynthesis pathway in *E. coli*. Inhibition pathways include feedback inhibition **(A)**, transcriptional inhibition **(B)**, and transport weakened **(C)**. Activities of three isoenzymes (*aroG*, *aroF*, and *aroH*) are inhibited by L-Phe, L-Tyr, and L-Trp. *TyrR* and *TrpR* suppress the expression of several crucial genes in the l-tryptophan biosynthesis pathway and l-tryptophan transport system. The right side shows derepression regulation mechanisms. The red solid line upward arrow indicates over-expression, the black solid line downward arrow indicates down-regulation, and × indicates knockout.

The SK pathway starts with DAHP, and 3-Deoxy-D-Arabino-Heptulosonate 7-Phosphate Synthase (DAHPS) is the first rate-limiting enzyme in this pathway. DAHPS in *E. coli* comprises three isoenzymes encoded by genes *aroG*, *aroF*, and *aroH*, which catalyze 79%, 20%, and 1% of DAHPS activity, respectively. Activities of these three isoenzymes are inhibited by L-Phe, L-Tyr, and L-Trp (Helmstaedt et al., 2005) (Figure 2). The construction of *aroG* ^*fbr*^ and *aroF* ^*fbr*^ mutants in the WF1234 strain lacking *aroB*, attenuated the feedback inhibition of isoenzymes by L-Phe and L-Tyr. The constructed mutant strain also overexpressed *tktA* and increased the net carbon flux from CCM to SK twice (Lin et al., 2014; Tyagi et al., 2017). An *aroG* variant resistant to L-Phe can be constructed by mutating the *SerA* at position 180. Eliminating the feedback inhibition of L-Phe could ultimately increase L-Trp production by 38.5% (Doroshenko et al., 2010; Chen et al., 2019). By reducing the copy number of L-Trp hydroxylated plasmids and replacing the promoter of the *aroH* ^*fbr*^ gene encoding *DAHPS*, the L-Trp conversion to 5-Hydroxytryptophan (5-HTP) in the recombinant strain *TRPmut/pSCHTP-LMT* was increased by 24.8%, compared to the original strain (Xu et al., 2020). Deregulating the natively regulated wild-type trp operon resulted in a maximum L-Trp yield of 40 g/L in batch fermentation (Chen and Zeng, 2017).

There are two isoenzymes of *DAHPS* in *S. cerevisia*e encoded by *ARO3* and *ARO4* genes. However, both isoenzymes face feedback inhibition by L-Phe and L-Tyr (Chrzanowski, 2020). The promoters of the genes involved in amino acid biosynthesis are usually regulated by *Gcn4* (Lou et al., 2022). However, the transcriptional effects of *Gcn4* on *ARO3* and *ARO4* genes are different under amino acid starvation. While *Gcn4* reduces the expression levels of *ARO3*, there are no inhibitory effects on the expression of *ARO4*. The feedback-resistant mutant *ARO4*-K229L causes the *DAHPS* to be insensitive to L-Phe and L-Tyr. Furthermore, reduced expression of *ARO3* gene in *ARO4*-K229L mutant can increase the SK flux by 4–5 times (Luttik et al., 2008a). In *E. coli*, several reactions in the SK pathway are catalyzed by individual enzymes.

On the contrary, in *S. cerevisiae*, conversion reactions are mainly carried out by the action of a five-function enzyme encoded by *ARO1* (Kuplińska and Rząd, 2021). The final step of the SK is catalyzed by *ARO2*-encoded chorionic synthase. After overexpression of *ARO2* in *S. cerevisiae*, the extraction of aromatic compounds from tyrosine resulted in a 2-folds increase in the yield of coumaric acid (Mao et al., 2017). Thus, overexpressing *ARO2* is advantageous to raise CHO levels. L-Tyr and L-Phe will gradually accumulate more due to the CHO pathway’s increased carbon flow, which will rise with the increased carbon flow. On the other hand, DAHPS feedback inhibition is an inevitable consequence of an excessive build-up of L-Trp. Therefore, it is essential to remove the feedback inhibition of *DAHPS* by L-Phe, L-Tyr, and L-Trp to facilitate the higher productivity of L-Trp in the SK pathway.

A study of the key genes involved in the production of DAHP in *E. coli* Trp01 indicated that the *aroF*, *aroG*, and *aroH* were considerably in the logarithmic and steady phase, indicating that separate enzymes catalyzed PEP and E4P. The new strain Trp07 produced 3.94 g/L of DAHP in shake flasks when the S180F mutation was added to the *aroG* gene to eliminate feedback inhibition of DAHP by L-Trp (Tang et al., 2023) and through the introduction of *ARO3* with *ARO4*-K229L, which is insensitive to *DAHPS*, in *S. cerevisiae*, overexpression of the *ARO4*-K229L allele increased the total flux of branched aromatic amino acid metabolism 3 to 4 times in comparison to the control bacterium, CEN. PK (Luttik et al., 2008b; Yang et al., 2021).

### Regulation of chorismate pathway

In *E. coli*, a part of the CHO pathway synthesizes L-Trp. The remaining reactions of the pathway are catalyzed by the Chorismate mutase (CM) to generate prebenzic acid (PPA). Furthermore, PPA is subsequently transformed into L-Phe and L-Tyr by prephenate dehydrogenase (encoded by *tyrA*) and prephenate dehydratase (encoded by *pheA*). By knocking out the *pheA* gene involved in the competing pathway encoding the Chorismate mutase-prephenate dehydrogenase (CM-PDT), the carbon flow to L-Tyr and L-Phe is blocked. In contrast, the carbon flow to the L-Trp producing reactions indirectly increases (Patnaik et al., 2008). Anthranilic acid (ANTA) synthase catalyzes the formation of ANTA from CHO, which enters the CHO pathway for L-Trp biosynthesis. ANTA synthase is encoded by the gene *trpED*, and L-Trp inhibits the enzyme activity of ANTA synthase. Gene transcription in the *trpEDCBA* operon is inhibited by *TrpR*. When the co-inhibitor L-Trp is present in large quantities, *trpR* and *tyrR* are active. *TyrR* and *TrpR* suppress the expression of several crucial genes in the L-tryptophan biosynthesis pathway and L-tryptophan transport system. Thus, these two repressors are typically detected concurrently or separately to improve L-tryptophan production (Jing et al., 2018) (Figure 2). Knocking out *trpR* and *tnaA* can remove the inhibitory effects of repressor protein and the degradation of tryptophan. Both *trpR* knockout strains and double-gene (*trpR* and *tnaA*) knockout strains produced a higher quantity of tryptophan (10- and 20-folds, respectively) (Yu et al., 2008). The inactivation of tryptophan attenuators and modification of the tryptophan operon promoter significantly improved L-Trp biosynthesis, and the engineered *E. coli* strain *GPT1002* produced 15.48 g/L of L-Trp in 10 h (Gu et al., 2012). Constitutive plasmids containing tryptophan operons (*trpE* ^*fbr*^, *trpD* ^*fbr*^) were transformed into JB102 strains lacking *trpR* and *tnaA* genes. After 27 h of fermentation, 6.2 g/L of L-Trp was produced by the JB102 strain (Dodge and Gerstner, 2002). After multiple mutagenesis breeding and adding surfactant L61, the L-Trp yield reached 54.5 g/L.

The mechanism of the CHO pathway in *S. cerevisiae* and *E. coli* is similar, and the generation of L-Phe and L-Tyr inhibits CHO in both microbes. To obtain the target product L-Trp, *ARO7* ^*G141S*^ must be overexpressed, increasing the anti-feedback inhibition of *DAHPS* (Cao et al., 2020). Genes involved in amino acid biosynthesis are widely distributed in yeast genomes, and both *Trp2* and *Trp3* contain binding sites for *Gcn4* (Mittal et al., 2017). Three *Gcn4* binding sites have been found in *Trp4*; one can also bind to the transcriptional regulator Pho2 (also known as Bas2) to participate in phosphate Pi metabolism (Cao et al., 2020). In phosphate Pi metabolism, PRPP is used as a *Trp4* substrate. When Pi is limited in the case of amino acid starvation, *Trp4* expression is determined by the Pho2, which occupies the binding site. Consequently, *Trp4* cannot bind with *Gcn4* to fully activate *TRP4* expression. Thus, Pho2 prevents the production of excess anthranilate phosphoribosyl transferase (Perveen et al., 2017). ANTH is the first intermediate in the tryptophan biosynthesis pathway, produced by *Trp2/Trp3* complexes in an amide group reaction (Blank et al., 2005).

The feedback-resistant DAHP synthase encoded by *aroF* was produced by eliminating its residue Ile11. For the production of a feedback-resistant ANTA synthase encoded by *trpED*, the substitution of the Ser40 residue with Phe was performed. The inhibition of the transcriptional regulation of the trp repressor and the degradation process of L-Trp were achieved by suppressing the *trpR* and *tnaA*-producing genes, respectively. By eliminating their respective essential genes, *pheA* and *tyrA*, two competing pathways that produce L-Phe and L-Tyr, were also inhibited (Zhao et al., 2011a). Contrarily, *TyrR* and the tryptophan manipulator are absent in *S*. *cerevisiae*. As a result, the carbon flux from the chorismate to ANTA can only be boosted by overexpressing *Trp2* and *Trp3* to increase the production of L-Trp (Kuivanen et al., 2021).

### Regulation of the L-Trp transport system

Research has indicated that there is an ongoing rise in intracellular tryptophan synthesis, which may be attributed to the reuptake and accumulation of specific tryptophan molecules by tryptophan isozyme or the retrieval of excreted tryptophan from the surrounding environment (Li et al., 2020b). During the fermentation, intracellular L-Trp is secreted extracellularly. Therefore, the L-Trp transport system must be modified to improve tryptophan production. Both *TnaB* and *Mtr* are specific tryptophan osmozymes. While *TnaB* is a low-affinity transporter encoding the *TnaA* operon, *Mtr* is a high-affinity tryptophan isozyme (Figures 1, 2) (Koyanagi et al., 2004). In the *E. coli* S015 strain, the expression of *tnaA* in the degradation pathway was blocked, and tryptophan-specific transporters *mtr* and *tnaB* were knocked out (van der Stel et al., 2021). Afterward, two feedback resistance genes, *aroG* ^*S180F,*^ and *SerA* ^*H344A/N364A*^ were generated by site-directed mutagenesis. Subsequently, *aroG* ^*S180F*^ and *SerA* ^*H344A/N364A*^ were linked to a strong ribosomal binding site (rbs, AAGGAG) and controlled by the strong promoter tac. An enhanced operon (PJ23119-rpsL-tac-aroG^S180F^-SerA^H344A/N364A^) was formed in the plasmid strp015A, and the yield of L-Trp was increased to 40.3 g/L (Chen and Zeng, 2017). Furthermore, the robust promoter trc was employed to substitute the regulatory region of the aforementioned operons for the purpose of regulating the L-Trp branching pathway, enhancing inhibition and attenuation, and attaining consistent overexpression of L-Trp operons (Du et al., 2019). It was found that the naturally regulated wild-type L-Trp operon in *E. coli* S019 was released, and the L-Trp yield increased to 2.14 ± 0.03 g/L. In another study, Ptrc-trpE and Ptrc-aroG were inserted into the *trpE* and *tyrR* locus. Consequently, feedback inhibition of *ANTHS* by *tyrR* was alleviated and 49 g/L L-Trp was accumulated after 36 h of fermentation in a 5 L bioreactor (Du et al., 2019).

AroP in *E. coli* TRP1 strain is a universal aromatic amino acid osmotic enzyme encoded by the *aroP* gene. aroP can also transport L-Phe and L-Tyr with high affinity. By knocking out the *aroP* gene and overexpressing the *yddG* gene in *E. coli* TRTH ΔaroP mutant, the L-Trp yield increased by 13.3% compared to the original strain (Liu et al., 2012). Increasing the efflux of L-Trp is an effective strategy for reducing the intracellular concentration of L-Trp and enhancing the glucose consumption rate. Nevertheless, these procedures cannot completely mitigate the feedback inhibitory effect of elevated levels of intracellular L-Trp. Hence, additional investigation is warranted to regulate the feedback-inhibitory impacts of L-Trp to enhance its production (Table 1).

**TABLE 1:** Comparison of industrial production strategies of L-Trp in *S. cerevisiae* and *E. coli*.

Pathway	Host	Strategy	Result	Reference
Central metabolism	*E. coli*	The construction of *PTS* mutant strains (*ptsHIcrr* ^*-*^ */glf-glk* ^*+*^and *ptsG* ^*-*^)	L-tryptophan production increasing 25.9% and 9.4%, respectively	Wu et al. (2017)
		The weak promoters *P*_*M1-12*_, *P*_*trc*_ *-pyc* ^*P458S*^ overexpressed *glf*, *glk* corresponding to strain SX11	The constructed strain SX11 produced 41.7 g/L L-Trp with an overall yield of 0.227 g/g glucose	Xiong et al. (2021)
		Overexpressing *pps* and/or *tktA*	2 times higher production of DAHP	Patnaik and Liao (1994)
		Upregulating *citT*	Accumulated 7 g/L L-Trp	Du et al. (2019)
		Knocking out the gene encoding PEP to degrade pyruvate kinases *pykA* and *pykf*	Increasing DAHP production by 3 times	Berry (1996), Gosset et al. (1996)
	*S. cerevisiae*	Overexpressing transketolase isoenzyme 1 (*Tkl1*), and deletion of *Zwf1*	Increasing the 3-DHS titer by 8.3 times	Curran et al. (2013), Deaner and Alper (2017)
		Expressing the *ARO4*-K229L (*ARO4* allele)	Resulting in the accumulation of 17.9 mg/g glucose and 358 mg/L DAHP	Suástegui et al. (2016)
SK	*E. coli*	The construction of *aroG* ^*fbr*^ and *aroF* ^*fbr*^ mutants and overexpressed *tktA* in the WF1234 strain lacking *aroB*	Increasing the net carbon flux from CCM to SK by 2 times	Lin et al. (2014), Tyagi et al. (2017)
		An *aroG* variant resistant to L-Phe was constructed by mutating the *SerA* at position 180	Increasing L-Trp production by 38.5%	Doroshenko et al. (2010), Chen et al. (2019)
		Reducing the copy number of L-Trp hydroxylated plasmids and replacing the promoter of the *aroH* ^*fbr*^ gene encoding DAHPS	The L-Trp conversion to 5-HTP in the recombinant strain *TRPmut/pSCHTP-LMT* was increased by 24.8%	Xu et al. (2020)
		Deregulating the natively regulated wild-type trp operon	L-Trp yield up to 40 g/L in batch fermentation	Chen and Zeng (2017)
	*S. cerevisiae*	Reducing expression of *ARO3* gene in *ARO4*-K229L mutant	Increasing the SK flux by 4–5 times	Luttik et al. (2008a)
		Overexpression of *ARO2*	Resulting in a 2-folds increase in the yield of coumaric acid	Mao et al. (2017)
CHO	*E. coli*	Both *trpR* knockout strains and double-gene (*trpR* and *tnaA*) knockout strains	Producing higher quantity of tryptophan (10- and 20-folds, respectively)	Yu et al. (2008)
		The inactivation of tryptophan attenuators and modification of the tryptophan operon promoter	The engineered *E. coli* strain GPT1002 produced 15.48 g/L of L-Trp	Gu et al. (2012)
		Constitutive plasmids containing tryptophan operons (*trpE* ^*fbr*^, *trpD* ^*fbr*^) were transformed into JB102 strains lacking *trpR* and *tnaA* genes	L-Trp yield reached 54.5 g/L via multiple mutagenesis breeding and the addition of surfactant	Dodge and Gerstner (2002)
	*S. cerevisiae*	Overexpression of *ARO7* ^*G141S*^	Increases the anti-feedback inhibition of DAHPS	Cao et al. (2020)

L-Trp is degraded by the serotonin pathway and kynurenine pathway. The serotonin pathway is initiated by tryptophan hydroxylase, and the 5′hydrogen atom on the tryptophan benzene ring is replaced by a hydroxyl group to produce 5-HTP. 5-HTP is further converted into melatonin, the final product of the serotonin pathway (Barik, 2020). On the other hand, the kynurenine pathway is initiated by one of two heme-containing oxidoreductases, indole 2,3-dioxygenase (IDO) or the highly related tryptophan 2,3-dioxygenase, producing kynurenine and niacin as primary metabolites (Munn and Mellor, 2013). IDO serves as the initial enzyme that restricts the pace of the metabolic process known as the kynurenine pathway. IDO was previously hypothesized to function as a modulator of the inflammatory response. The subsequent research demonstrated that immune cells that express IDO1, such as macrophages and dendritic cells, exerted an inhibitory effect on the proliferation of T cells (Hwu et al., 2000; Frumento et al., 2002; Munn et al., 2002). It has also been shown that exogenously added kynurenine binds to transforming growth factors and acts as an immunosuppressive metabolite (Orabona and Grohmann, 2011). L-Trp regulates gluconeogenesis to maintain blood sugar levels and prevent hypoglycemia (Badawy, 2017).

## The industrial production of L-Trp

In the industrial setting, microbial fermentation is widely employed to create L-Trp and synthesize several derivatives. Researchers are interested in increasing the industrial yield of L-Trp by examining potential metabolic regulatory networks of L-Trp biosynthesis and altering them. Because of their high output and easier regulation of the L-tryptophan biosynthesis pathway, *E. coli* and *C. glutamicum* are frequently utilized as hosts for L-Trp production (Liu et al., 2016). The industrial production of L-Trp in *E. coli*, *C. glutamicum*, and *S*. *cerevisiae* is summarized (Table 2).

**TABLE 2:** Summary of industrial production of L-Trp.

Host	Strain	Engineering strategy	Study details	Improvement	Reference
*E. coli*	TS-1	By enhancing the flow of cofactors and precursors used in biosynthesis	Overexpressing *icd*, *gdhA*, *prs*, *serA*, *thrA*, syhA and *pntAB*	+2.76-times higher than the L-Trp titer of KW001 (from 1.380 g/L to 1.710 g/L)	Li et al. (2020b)
	TRTH0709/pMEL03	By decreasing the associated biosynthetic pathway’s feedback inhibition	Overexpressing *yddG* and reducing the activity of the *pta-mtr*	+15.96% higher than the parental strain (L-Trp titer up to 48.68 g/L)	Wang et al. (2013)
	GPT2000	By enhancing the supply of precursors	Introduction of the *phaCAB* manipulator gene, upregulation of the transcription of the tryptophan manipulator	+1.9 to 4.3 times more than the control (L-Trp titer up to 14.4 g/L)	Gu et al. (2013)
	FB-04/pSV03	The genome’s use of gene modification techniques	By overpressing feedback-resistant genes for *AroF* and *TrpED*, deleting the tryptophan repressor	+10% conversion ratio from glucose (L-Trp titer up to 13.3 g/L)	Zhao et al. (2011b)
	FB-04 (*pta*1)	By increasing carbon flux in metabolic pathways	Knockdowning *pta*	+15% increase over that of FB-04	Liu et al. (2016)
	TRP07	By modifying the central metabolic pathway	Expressing the coding genes *acnBA* and *icd* in tandem regulated by *PacnB*	−0.8 g/L and 2.2 g/L of the by-products	Du et al. (2019)
	S028	Using targeted molecular engineering approaches	Increasing the initial inoculum of the strain S028	+2-folds as much as other L-Trp producers	Chen and Zeng (2017)
	S092	By feed-forward regulation	Expressing the anthranilate-activated *TrpC* from *A. niger*	+1.5-folds of L-Trp (from 19 g/L to 29 g/L)	Chen et al. (2018a)
	TPR9	Utilizing modular engineering strategies	Strengthening the expression of L-Trp operon and optimizing the key genes *serA* and *prs*	+81.7% of the maximum theoretical yield	Ding et al. (2023)
*C. glutamicum*	pKW9901	Developing a unique plasmid stabilization system	*PGD* was cloned from wild-type *C. glutamicum* ATCC 31833	+61% more than strain KY10894	Ikeda et al. (1994)
	pIK9960	By a plasmid stabilization system and plasmid-mediated amplification of the genes	Low-copy-number plasmid pSW9911 inserted the transketolase gene	+15% increase over the titers of the pKW9901 and pSW9911	Ikeda and Katsumata (1999)
	ACGEB2b	UV radiation-induced mutations	UV radiation	+1,257.4% increment	Mohamed et al. (2018)
	ACGEB2c			+295% increment	
*S. cerevisiae*	ST7574 (CEN.PK113-7D)	Engineering the industrial workhorse *S. cerevisiae*	With *SrPyrH*, *SttH*, and *LaRebH*	+3.56 ± 0.24 mg/L 7-bromotryptophan	Milne et al. (2023)

After 48 h of fermentation in shake flasks, the industrial strain of *E. coli* TS-1 produced 1.710 g/L L-Trp due to the overexpression of *icd* and *gdhA*, the addition of *prs*, the re-expression of mutant *serA* and *thrA* to increase the supply of serine precursors, and eventually the overexpression of *syhA* and *pntAB* (Li et al., 2020b). In *E. coli* TRTH0709/pMEL03, overexpression of *yddG* and suppression of phosphate acetyltransferase-high affinity tryptophan transporter (*pta-mtr*) led to L-Trp titers of up to 48.68 g/L when batch fermentation was added after 48 h in 30 L fermenter (Wang et al., 2013). It was discovered that PHB could upregulate the transcription of the tryptophan manipulator by producing L-Trp-producing *E. coli* GPT1002, into which the PHB biosynthesis pathway containing the *phaCAB* manipulator gene was introduced. Xylose was added as a co-substrate to the medium to increase the supply of precursors for PHB biosynthesis. By replenishing batch culture, 14.4 g/L of L-Trp was produced in GPT2000 (Gu et al., 2013) by overpressing feedback-resistant genes for *AroF* and *TrpED*, deleting the tryptophan repressor, and preventing the manufacture of L-Phe and L-Tyr. The finished modified *E. coli* FB-04/pSV03 can generate 13.3 g/L of L-Trp (Zhao et al., 2011b). After 55 h of batch fermentation with supplementation, the final L-Trp titer was 44.0 g/L due to the phosphate acetyltransferase knockdown in *E. coli* FB-04 (Liu et al., 2016; van der Stel et al., 2021). By co-expressing the coding genes *acnBA* and *icd* under the control of *PacnB*, the TRP07 collected 49 g/L L-Trp after 36 h in a 5 L bioreactor (Du et al., 2019). The L-Trp concentration was increased up to 40.3 g/L by augmenting the initial inoculum of the S028 strain. The expression of anthranilate-activated *TrpC* from *A. niger* in the S092 resulted in a significant increase in L-Trp concentration, elevating it from 19 to 29 g/L within 42 h (Chen et al., 2018a). After fermentation in a 5 L bioreactor for 40 h, the high-yield strain TRP9 achieved an L-Trp yield of 36.08 g/L by improving the expression of the L-Trp operon and optimizing the essential genes *serA* and *prs* for the synthesis of serine and PRPP in the chorismate to L-Trp module (Ding et al., 2023).

The gene for 3-phosphoglycerate dehydrogenase (PGD), the first enzyme in the serine pathway, was cloned from wild-type *C. glutamicum* ATCC 31833 and linked onto pKW99 to generate pKW9901. This increased the amount of carbon flow into serine. The best recombinant strain thus created can produce L-Trp at a titer of 50 g/L after 80 h of growth in jar fermentors without antibiotics (Ikeda et al., 1994). The final L-Trp titer for pIK9960 was 58 g/L, 15% higher than for carriers pKW9901 and pSW9911 (Ikeda and Katsumata, 1999). UV radiation of the ACGEB1 and ACGEB2 *C. glutamicum*. L-Trp levels before mutation in *C. glutamicum* ACGEB1 were 19.6 g/mL and 20.48 g/mL in ACGEB2, respectively. Particularly in C.G. ACGEB2b (278.4 g/mL), followed by C.G. ACGEB2c (81.6 g/mL), the L-Trp output is noticeably boosted (Mohamed et al., 2018).

L-tryptophan synthesis in *S. cerevisiae* for using in industry has been documented less frequently and with lower yields. It was discovered that engineering *S. cerevisiae* for the *de novo* production of Halogenated Tryptophan and Tryptamine Derivatives (Gong et al., 2022). Integration of *S. cerevisiae* ST7574 (CEN.PK113-7D) with *SrPyrH*, *SttH*, and *LaRebH*. The highest titers obtained for halogenated tryptophan and tryptamine were 3.56 ± 0.24 mg/L 7-bromotryptophan and 2.42 ± 0.31 mg/L 7-chlorotryptamine (Milne et al., 2023).

Metabolic engineering strategy has significantly increased the production of L-Trp by introducing new pathways and rewiring native metabolism. However, a number of unfavorable factors significantly restrict microbial growth and metabolic activity during the fermentation process (Mohedano et al., 2022). Therefore, improving the robustness and tolerance of microbial cell factory is essential to fulfill the requirements of industrial applications and gain maximum economic benefits (Gong et al., 2017). Numerous strategies, such as the most used adaptive laboratory evolution, have been developed and employed to study stress tolerance mechanisms and develop robust industrial *E. coli* and *S. cerevisiae* (Pereira et al., 2019). Recent academic research have suggested that the metabolism of amino acids may improve the resilience of microbes to stress (Horinouchi et al., 2010; Ohashi and Chaleckis, 2021). Therefore, regulation of amino acids may provide a novel strategy to improve the microbial ability to ferment in challenging fermentaion conditions.

## L-Trp regulation reform *S. cerevisiae* stress fitness

**FIGURE 3:**
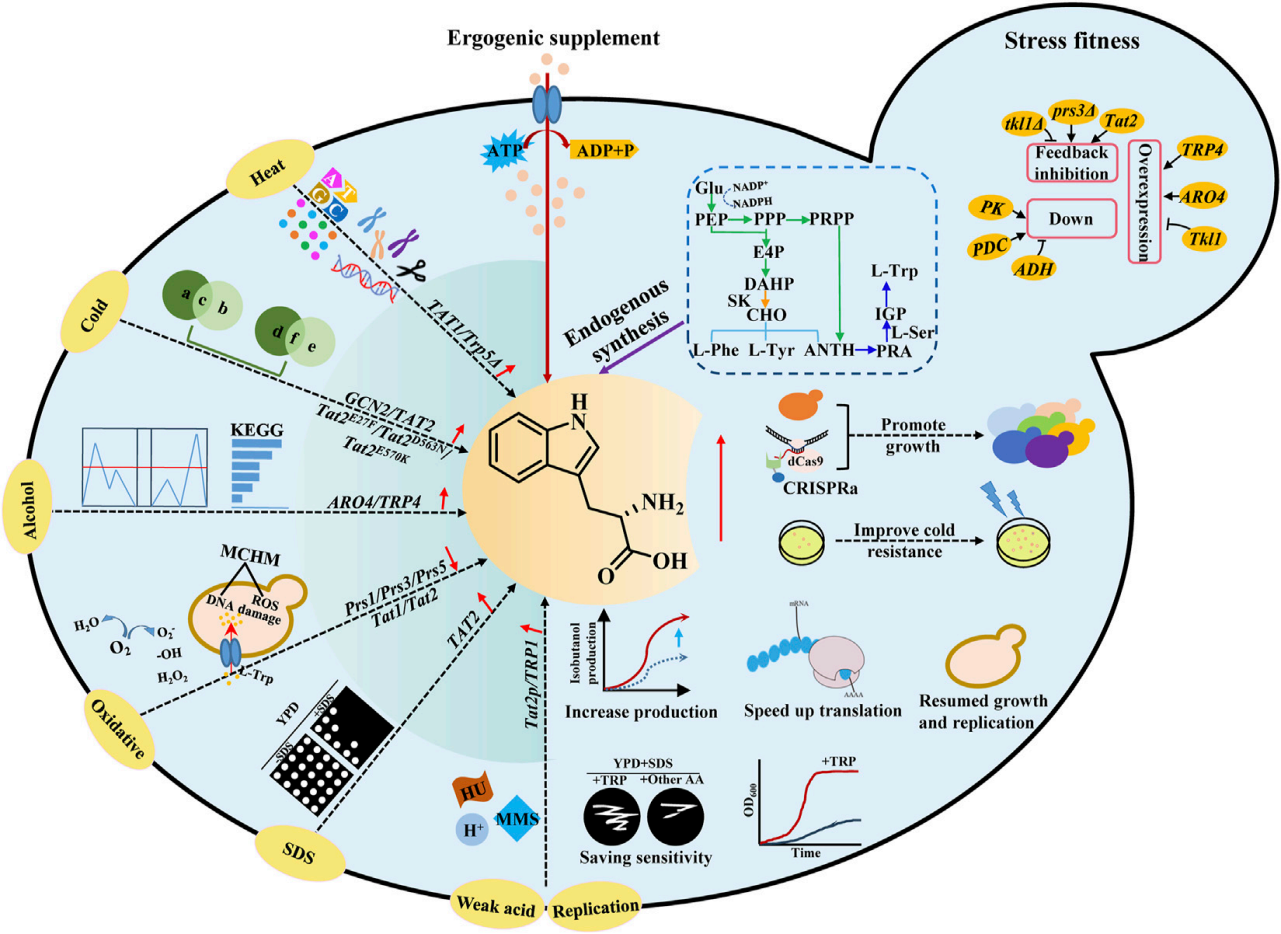
Effects of L-Trp regulation on stress fitness of *S. cerevisiae*. *S. cerevisiae* often faces various environmental stresses, including extreme temperature, ethanol, oxidation, and replication stress. Stress fitness of *S. cerevisiae* could be improved by exogenous supplement and endogenous synthesis of L-Trp. *TAT1* and *TAT2* encode tryptophan amino acid transporters. *TRP1*, *TRP2*, *TRP4*, and *TRP5* are key genes for L-Trp biosynthesis. *ARO4* encodes DAHP synthase, catalyzes the first step in aromatic amino acid biosynthesis, and is feedback-inhibited by high concentrations of L-Trp. *PRS1*, *PRS3*, and *PRS5* encode ribose phosphate diphosphokinase subunits. *GCN2* encodes serine/threonine-protein kinase.

*S. cerevisiae* is widely used to produce biochemical products, biofuels, high-value natural products, and fermented foods. During the fermentation process, *S. cerevisiae* often faces various environmental stresses, including extreme temperature, ethanol, oxidation, and replication stress (Deparis et al., 2017; Shen et al., 2022) (Figure 3). In recent years, academic studies have noted a notable focus on investigating the relationship between amino acid metabolism and stress fitness. In the following subsections, the effects of L-Trp on the stress tolerance of yeast have been summarized (Figure 3; Table 3).

**TABLE 3:** The relationship between L-Trp and stress fitness in *S. cerevisiae*

Stress	Strategy	Stress fitness	Reference
Temperature stress			
Heat	Overexpressing the *amino acid transporter TAT1*	Increase	Palmer et al. (2002)
	Supplementing 10 μM L-Trp in SD medium for *trp5*Δ	Increase	Jarolim et al. (2013)
	Supplementing high L-Trp and leucine during heat shock treatment	Increase	Luethy et al. (2020)
Cold	Overexpressing the tryptophan permease *TAT2* in tryptophan deficient strain YPH499	Increase	Abe and Horikoshi (2000)
	The N- and C-terminal mutations in tryptophan permease Tat2 (Tat2^E27F^, Tat2^D563N^, Tat2^E570K^)	Increase	Nagayama et al. (2004)
Alcohol stress			
Ethanol	Overexpressing of TRP1, TRP2, and TRP3	Increase	Hirasawa et al. (2007)
	Upregulation of differentially expressed genes (DEGs) and differentially expressed proteins (DEPs) associated with aromatic amino acids, such as *ARO2*, *ARO4*, *ARO9*, and *TRP4*	Increase	Li et al. (2019)
Isobutanol	Constructing *S. cerevisiae* strains by deleting the TRP genes	Increase	Kuroda et al. (2019)
	Adding exogenous tryptophan to the growth medium	Increase	Liu et al. (2021)
Oxidative stress			
ROS	Synthesizing self-healing substances, such as tryptophan	Increase	Ayers et al. (2020)
	The expressions of genes involved in amino acid synthesis and transportation, such as *Prs1*, *Prs3*, *Tat1*, and *Tat2*	Decrease	Hernando et al. (1999)
Other coercive			
SDS	Overexpressing *TAT2* tryptophan isoenzyme	Increase	Schroeder and Ikui (2019)
Weak acid	Overexpressing *Tat2p* high-affinity tryptophan permease	Increase	Bauer et al. (2003)
Replication	Overexpressing *TRP1*	Decrease	Polleys and Bertuch (2015)

### Temperature stress fitness

The suitable temperature for the growth and metabolism of *S. cerevisiae* is 30°C–32°C. *S. cerevisiae* uses complex regulatory mechanisms to respond to temperature stress. One such mechanism involves the regulation of tryptophan metabolism (Pan et al., 2019; Shen et al., 2020; Choo et al., 2021). *S. cerevisiae* commonly encounters high-temperature stress during the process of fermentation. According to reports, the heat-shock response is activated in the *trp5*Δ strain, which is deficient in tryptophan synthesis due to starvation. Additionally, it has been observed that mutants with impaired L-Trp metabolism exhibit resistance to heat stress during both growth stages (Jarolim et al., 2013). As compared to standard Synthetic Complete (SC) media (composed of 20 mg/L tryptophan and 100 mg/mL leucine), high tryptophan and leucine supplementation during heat shock treatment resulted in >50% reduction in Tribromoethanol-mediated thermotolerance (Luethy et al., 2020).

Yeast cells frequently experience low-temperature stress during the accumulation of volatile compounds. However, low-temperature stress may result in a reduced growth rate and increased energy consumption (Xu et al., 2022). For tryptophan auxotrophic yeast, tryptophan appears to be the primary limiting factor for low-temperature resistance (Lopez-Malo et al., 2014). Eliminating the *TRP1* gene, which encodes a phospho-ribosyl anthranilate isomerase involved in tryptophan biosynthesis, increases susceptibility to low temperatures (Ballester-Tomás et al., 2017). However, overexpression of the tryptophan permeases *TAT1* and *TAT2*, also known as *SCM2*, can enhance the cold tolerance of yeast (Vicent et al., 2015; Kuroda et al., 2019). The tryptophan auxotrophic strain YPH499 could transport tryptophan-containing oligopeptides via its peptide transporter to compensate for the inability to grow at low temperatures (Kitagawa et al., 2013). High levels of Tat2 protein (a high-affinity tryptophan permease) expression resulted in cells having the capacity to grow at low temperatures of 10°C–15°C (Abe and Horikoshi, 2000). In addition, the N- and C-terminal mutagenesis of *HPG2* (a *TAT2* allele) improved Tat2 stability during low-temperature incubation, leading to cell growth under these demanding circumstances (Nagayama et al., 2004).

### Alcohol stress fitness

The most famous application of *S. cerevisiae* is to produce bioethanol using biomass resources such as cellulose and starch. However, high levels of ethanol affect the growth and productivity of yeast cells. Ethanol concentrations above 9% (v/v) have been reported to influence the development of *S. cerevisiae* cells (Li et al., 2019). Excessive concentrations of alcohols mainly affect cell membrane integrity, vacuole structure, and function (Stanley et al., 2010; IngoLFSSON Helgi and Andersen Olaf., 2011). Ethanol stress tolerance-related gene in *S. cerevisiae* was studied based on DNA microarray, and yeast cells showed tolerance to 5% ethanol after overexpression of *TRP1*, *TRP2*, and *TRP3* (Hirasawa et al., 2007). Upregulation of differentially expressed genes (DEGs) and differentially expressed proteins (DEPs) associated with aromatic amino acids, such as *ARO2*, *ARO4*, *ARO9*, and *TRP4*, is conducive to the upregulation of *TRP4* genes, especially under ethanol stress. Consequently, upregulating DEGs and DEPs facilitates the catalysis of tryptophan synthesis and serves as a protective mechanism against ethanol-induced stress in *S. cerevisiae* cells. This protective mechanism is achieved by creating hydrophobic areas, which ultimately contribute to the survival of the cells, as mentioned above (Li et al., 2019).

Isobutanol is also considered a potential biofuel, and *S. cerevisiae* is one of the most suitable hosts to produce isobutanol (Wess et al., 2019). However, branched-chain alcohols may cause severe cytotoxic effects by disrupting the cell membranes, thus becoming a prominent barrier during isobutanol production (Kuroda et al., 2019). Kuroda et al. (2019) produced *S. cerevisiae* strains by eliminating the *TRP* genes, which encode tryptophan production enzymes. Isobutanol sensitivity is raised in the altered strains. Moreover, the regulation of tryptophan metabolism improved the isobutanol tolerance, thus increasing the yield of isobutanol in engineered *S. cerevisiae* strains (Kuroda et al., 2019). Liu et al. (2021) restored the isobutanol tolerance in the damaged strains by adding exogenous tryptophan to the growth medium, demonstrating the significance of tryptophan in improving the isobutanol tolerance in yeast (Liu et al., 2021).

Moreover, transcriptome studies demonstrated that in conditions similar to nitrogen deficiency, the genes involved in tryptophan synthesis and transportation were increased (Liu et al., 2021). In summary, tryptophan and associated synthetic regulatory networks are critical in yeast’s ability to withstand alcohol stress.

### Oxidative stress fitness

Reactive oxygen species (ROS), the partially reduced forms of molecular oxygen, are the primary cause of oxidative stress in all aerobically growing organisms. High levels of oxidative stress may damage cellular constituents, such as DNA, lipid, and protein lesions (Alves et al., 2023). To cope with ROS, yeast cells quickly adjust and synthesize self-healing substanceslike tryptophan. Other than malnutrition, L-Trp has been suggested to be connected to stress tolerance (Ayers et al., 2020). The expressions of genes involved in amino acid synthesis and transportation, such as *Prs1*, *Prs3*, *Tat1*, and *Tat2*, must be interrupted to weaken the metabolism of tryptophan and other amino acids. The proliferation of yeast cells experiences a state of stagnation due to inadequate nutrient availability. However, this growth inhibition can be alleviated by introducing supplementary tryptophan to the growth media. This supplementation facilitates reinstating a balanced distribution of nitrogen sources within the medium (Hernando et al., 1999).

### Other coercive effects

When exposed to different external pressures, yeast cells rely on cell walls and membranes as the primary protective barrier against these shocks. The activation of the multifactorial stress response in *S. cerevisiae* occurs when any disruption hinders the normal functioning of the cell wall or cell membrane (Levin, 2005; Levin, 2011). Sodium dodecyl sulfate (SDS) is a detergent that destroys the cell membranes (Cócera et al., 1999; Cócera et al., 2004), activates the cell wall integrity (CWI) signaling, and restricts the growth of *S. cerevisiae* cells (Igual et al., 1996; Bickle et al., 1998). Cell wall and plasma membrane integrity stress in yeast involving the *MCK1* effector is sensitive to SDS. This stress depends on tryptophan biosynthesis and levels of L-Trp and L-Tyr in media and cells (Schroeder and Ikui, 2019). In *S. cerevisiae*, *ΔMCK1* was used in the presence of SDS to screen inhibitory genes. The results showed that the overexpression of *TAT2* tryptophan isoenzyme resulted in higher L-Trp synthesis in cells, thereby restoring the growth of SDS-treated *ΔMCK1* cells.

*S. cerevisiae* can grow with weak organic acid preservatives, such as sorbate and benzoate, which also cause food and beverage deterioration (Piper et al., 2001). In the context of industrial utilization of *S. cerevisiae*, exposure to weak acid stress predominantly has deleterious consequences on cellular function by compromising the integrity of the cellular membrane. Additionally, this stress condition hinders yeast cells’ absorption of aromatic amino acids. The issue of weak acid hypersensitivity in the tryptophan biosynthesis pathway is resolved by introducing elevated concentrations of L-Trp into the growth medium. The overexpression of *Tat2p*, a high-affinity tryptophan permease, is employed to mitigate the sensitivity associated with sorbate (Bauer et al., 2003).

L-Trp is a precursor for the production of serotonin as well as NAD^+^. In *S. cerevisiae*, *Tat2* is sub-morphic at colder temperatures. After disrupting tryptophan biosynthetic genes, growth at room temperature induces tryptophan depletion (Abe and Horikoshi, 2000). To determine whether the L-Trp biosynthetic gene is involved in the resistance of yeast cells to replication stress, the sensitivity of *trp1-1* strain to DNA damaging agents, such as methyl mesylate (MMS) and hydroxyurea (HU), was detected (Godin et al., 2016). In contrast, the sensitivity of *trp1-1* strain to replication stress was inhibited by overexpressing *TRP1*.

Moreover, the yeast cells grew more slowly in the tryptophan-deficient medium at 23°C than at 28°C and 30°C. Therefore, the DNA damage sensitivity of *trp1-1* cells was specific for tryptophan biosynthesis. This proved that both low temperature and tryptophan consumption led to the sensitivity of cells to replication stress (Polleys and Bertuch, 2015). The study further suggested that the DNA damage sensitivity of L-Trp depletion might be due to impaired protein synthesis. However, DNA damage repair and L-Trp consumption are still being explored while applying L-Trp as a nutritional marker.

## Conclusion

With the continuous advancements in metabolic engineering and synthetic biology, green biosynthesis has gradually replaced L-Trp chemical synthesis. With the improvements in the anabolism of L-Trp, the systematic metabolic network of L-Trp in *E. coli* is more clearly understood. However, the mechanism of L-Trp biosynthesis and metabolic regulation in *S. cerevisiae* remains unclear and needs further exploration. In the CHO pathway, the knock out of branched acid isomerase inhibits the production of L-Phe and L-Tyr, and the efflux mechanism of L-Trp is critical for Trp production. Secondly, the associated citric acid pathway and TCA cycle play essential roles in the supply of precursors.

Further investigation is required to examine the accumulation of L-Trp in meticulously controlled settings, such as compromised L-Trp metabolic pathways. Furthermore, the proliferation of *S. cerevisiae* cells is influenced by various environmental stressors. Additionally, L-Trp is crucial for enhancing stress resistance in *S. cerevisiae*. On one hand, it can promote L-Trp synthesis or add exogenous L-Trp to play a “detoxifying” role. On the other hand, it can encourage yeast cell growth through L-Trp derivatives such as 5-HTP.

The L-Trp biosynthetic system still needs further expansion and improvement, especially in terms of metabolic regulation and engineering of *S. cerevisiae* to regulate some key enzymes and genes and inhibit the decomposition of L-Trp. The involvement of L-Trp in *S. cerevisiae*’s response to other stressful environments also needs to be explored further.

## Future considerations

Regulating tryptophan metabolism has been proven to be an efficient method to enhance the stress fitness of *S. cerevisiae*, especially the defective mutants of tryptophan synthesis. Thus, the regulation of tryptophan metabolism should be explored to develop novel strategies for producing stress-tolerant yeasts. Notably, the dosage of tryptophan in the media seems to be a crucial factor affecting the stress tolerance of yeasts. In recent research, exogenous supplementation of tryptophan has been commonly used to improve yeast stress tolerance. Furthermore, the development of dCas9-assisted enzyme perturbation provides a powerful tool for the graded expression of pathway enzymes. Compared to exogenous supplementation of tryptophan, regulating endogenous genes to synthesize appropriate quantity of tryptophan would be more cost-effective and stable. Thus, in further studies, the graded regulation of the tryptophan metabolism pathway needs to be explored to develop robust industrial cell factories. Previous results have signified that the external supply of critical amino acids may decrease cellular stress and improve process productivity in *E. coli* (Kumar et al., 2020). However, there are rare investigations to investigate the relationship between L-Trp metabolism and stress fitness in *E. coli*. In the future, the mechanism of tryptophan-mediated stress fitness should be clarified, which may provide novel insights to enhance the stress tolerance of yeast and *E. coli*.
